# Therapeutic Alliance and Rapport Modulate Responses to Psilocybin Assisted Therapy for Depression

**DOI:** 10.3389/fphar.2021.788155

**Published:** 2022-03-31

**Authors:** Roberta Murphy, Hannes Kettner, Rick Zeifman, Bruna Giribaldi, Laura Kartner, Jonny Martell, Tim Read, Ashleigh Murphy-Beiner, Michelle Baker-Jones, David Nutt, David Erritzoe, Rosalind Watts, Robin Carhart-Harris

**Affiliations:** ^1^ Department of Brain Sciences, Centre for Psychedelic Research, Imperial College London, Faculty of Medicine, London, United Kingdom; ^2^ Medical Psychotherapy, South West London and St. Georges NHS Trust, London, United Kingdom; ^3^ Department of Psychology, Ryerson University, Toronto, ON, Canada; ^4^ Medical Psychotherapy and General Adult Psychiatry, Devon Partnership NHS Trust, Exeter, United Kingdom; ^5^ Department of Psychology, Royal Holloway University, Surrey, United Kingdom; ^6^ Psychedelics Division Neuroscape, Department of Neurology, University of California, San Francisco, San Francisco, CA, United States

**Keywords:** psilocybin, therapeutic alliance, therapeutic relationship, emotional breakthrough, mystical experience, depression, acute psychedelic experience, psychedelic assisted therapy

## Abstract

**Background:** Across psychotherapeutic frameworks, the strength of the therapeutic alliance has been found to correlate with treatment outcomes; however, its role has never been formally assessed in a trial of psychedelic-assisted therapy. We aimed to investigate the relationships between therapeutic alliance and rapport, the quality of the acute psychedelic experience and treatment outcomes.

**Methods:** This 2-arm double-blind randomized controlled trial compared escitalopram with psychedelic-assisted therapy for moderate-severe depressive disorder (*N* = 59). This analysis focused on the psilocybin condition (*n* = 30), who received two oral doses of 25 mg psilocybin, 3-weeks apart, with psychological preparation, in-session support, and integration therapy. A new psychedelic therapy model, called “Accept-Connect-Embody” (ACE), was developed in this trial. The primary outcome was depression severity 6 weeks post treatment (Quick Inventory of Depressive Symptomatology, QIDS-SR-16). Path analyses tested the hypothesis that therapeutic alliance (Scale To Assess the Therapeutic Relationship Patient Version, STAR-P) would predict depression outcomes via its influence on the acute psychedelic experience, specifically emotional-breakthrough (EBI) and mystical-type experiences (MEQ). The same analysis was performed on the escitalopram arm to test specificity.

**Results:** The strength of therapeutic alliance predicted pre-session rapport, greater emotional-breakthrough and mystical-type experience (maximum EBI and MEQ scores across the two psilocybin sessions) and final QIDS scores (*β* = −0.22, *R* ^2^ = 0.42 for EBI_Max_; *β* = −0.19, *R* ^2^ = 0.32 for MEQ_Max_). Exploratory path models revealed that final depression outcomes were more strongly affected by emotional breakthrough during the first, and mystical experience during the second session. Emotional breakthrough, but not mystical experience, during the first session had a positive effect on therapeutic alliance ahead of the second session (*β* = 0.79, *p* < 0.0001). Therapeutic alliance ahead of the second session had a direct impact on final depression scores, not mediated by the acute experience, with a weaker alliance ahead of the second psilocybin session predicting higher absolute depression scores at endpoint (*β* = −0.49, *p* < 0.001)

**Discussion:** Future research could consider therapist training and characteristics; specific participant factors, e.g., attachment style or interpersonal trauma, which may underlie the quality of the therapeutic relationship, the psychedelic experience and clinical outcomes; and consider how therapeutic approaches might adapt in cases of weaker therapeutic alliance.

**Clinical Trial Registration:** This trial is registered at http://clinicaltrials.gov, identifier (NCT03429075).

## Introduction

Psychedelic-assisted therapy is a mental health intervention that involves the administration of a psychedelic substance, such as psilocybin, in combination with therapy or psychological support (Nutt and Carhart-Harris, 2021). Use of psychedelic substances within indigenous sacramental and healing rituals dates back thousands of years (Schultes et al., 2001). Psychedelic-assisted therapy was widely researched, and noted for its promise, during the 1950s and 60s, until it was shut-down due to restrictive governmental regulations (Nutt et al., 2020). Over the past decade, psychedelic-assisted therapy research has re-emerged with a growing body of evidence supporting its efficaciousness for a wide range of mental health presentations (for a review, see Andersen et al., 2021), including distress associated with a life-threatening illness (Grob et al., 2011; Griffiths et al., 2016; Ross et al., 2016; Anderson et al., 2020), substance misuse (Krebs and Johansen, 2012; Johnson et al., 2014, 2017; Bogenschutz et al., 2015), and major depressive disorder (MDD) (Carhart-Harris et al., 2016; Carhart-Harris L. et al., 2018; Palhano-Fontes et al., 2019; Carhart-Harris et al., 2021a; Davis et al., 2021). Most relevantly for the present work, in the recently published results of a double-blind randomised controlled trial (DB-RCT), analysed here in this paper, there was no significant difference found between the psilocybin-assisted therapy versus a treatment arm that featured 6 weeks of daily ingestion of a selective serotonin reuptake inhibitor (SSRI) antidepressant, escitalopram, in the primary outcome (change in QIDS from baseline to primary endpoint), with the same psychological support (Carhart-Harris et al., 2021a). However, we found more rapid and wide-ranging mental health improvements in the psilocybin arm. Given these findings, alongside the growing evidence base for the efficacy of psychedelic-assisted therapy, there is a need to better understand its therapeutic mechanisms of action.

Extant research suggests that the quality of the acute psychedelic experience is predictive of psychedelic-assisted therapy’s positive therapeutic effects (Roseman et al., 2018; Romeo et al., 2021). To date, the most extensively researched dimension of the acute psychedelic experience is the so-called ‘mystical-type’ or ‘peak’ experience, which includes deeply felt positive mood, ineffability, a sense of sacredness and unity, and transcendence (see Johnson et al., 2019). Several studies have provided evidence for the role of the mystical-type experience as a predictor of positive treatment outcomes following psychedelic-assisted therapy (Bogenschutz and Johnson, 2015; Carhart-Harris et al., 2018; Garcia-Romeu et al., 2014; Griffiths et al., 2016; Johnson et al., 2017; Roseman et al., 2018; Ross et al., 2016; for a review, see; Romeo et al., 2021). Beyond the mystical-type experience, recent prospective research (Roseman et al., 2019) and published accounts of trial participant’s psychedelic therapy experiences (Gasser et al., 2014; Watts et al., 2017) have also highlighted the importance of emotional breakthrough; i.e., experiencing emotional release or catharsis after facing and overcoming difficult or previously inaccessible emotions or memories, and gaining personal and interpersonal insights; therapeutic mechanisms well established within traditional psychotherapeutic schools (Foa and Kozak, 1986; Jackson, 1994; Samoilov and Goldfried, 2000; Greenberg and Pascual-Leone, 2006; Pascual-Leone et al., 2015). One of the proposed mechanisms of psychedelics is that they weaken ego boundaries and allow access to the ‘unconscious mind’ (Frederking, 1955; Grof, 2008), and in line with this, many psychedelic therapists have written about the importance of emotional breakthrough as a therapeutic mechanism (see Roseman et al., 2019 for a comprehensive background). Results of survey-based data of individuals planning to use a psychedelic suggested that higher levels of emotional breakthrough are associated with improved mental health in the weeks following psychedelic use (Roseman et al., 2019; Spriggs et al., 2021). Importantly, however, the role of emotional breakthrough in facilitating treatment outcomes has not yet been evaluated within a clinical sample or in the context of a controlled psychedelic-assisted therapy clinical trial.

Empirical research to date has focused primarily on the role of the acute psychedelic experience as a mediator of treatment outcomes, with relatively less focus on the role of other important components of psychedelic-assisted therapy that may serve to promote certain acute experiences supportive of clinical improvements. There is some evidence to support a strong and long-held assumption in psychedelic-assisted therapy research that psychological and clinical outcomes are critically dependent upon the surrounding context in which they are taken (Leary et al., 1963; Hartogsohn, 2016, 2017; Carhart-Harris and Nutt, 2017; Carhart-Harris et al., 2018). One key contextual component in this regard is the relationship between the individual receiving psychedelic-therapy and the treatment provider(s) i.e., the ‘guides’ or therapists (Grinspoon and Doblin, 2001; Grof, 2008; Johnson et al., 2008; Richards, 2016; Carhart-Harris and Nutt, 2017; Breeksema et al., 2020; Nayak and Johnson, 2020; Watts and Luoma, 2020). Across a range of cultural and historical contexts there has always been a strong emphasis on the importance of the guide, therapist or shaman in supporting psychedelic experiences (Walsh and Grob, 2005; Grof, 2008; Richards, 2016; George et al., 2020). In contemporary psychedelic research, these individuals are most often referred to as “guides” but can also be referred to as “sitters” or “therapists”. Similarly, the terms “participants” and “patients” tend to be used interchangeably depending on the specific context in psychedelic literature. Therapeutic approaches in psychedelic-assisted therapy are evolving and vary across different clinical trials, as does the emphasis on the importance of the role of the therapist or guides [see methods for details of the Accept Connect Embody (ACE; Watts and Luoma, 2020) therapeutic protocol], but typically these guides take a supportive, open and non-directive approach and aim to provide a safe and containing setting with a view to allowing unconscious psychological material, emotions, memories and difficulties to emerge (Grinspoon and Doblin, 2001; Grof, 2008; Breeksema et al., 2020; Nayak and Johnson, 2020; Watts and Luoma, 2020). The standard model is to allocate two such guides to each participant (Walsh and Grob, 2005; Grof, 2008; Richards, 2016), who are typically mental health professionals, ideally with personal and/or professional experience with the effects of psychedelic compounds or other non-ordinary states. In this trial participants had approximately 35–40 h of contact with their guide, 16 of which were the two psilocybin session days.

The therapeutic relationship, i.e. the relationship between therapist and patient, is recognised to be of fundamental importance across different psychotherapeutic models or approaches (Horvath, 2000; Lemma, 2015; Baier et al., 2020). Some particularly relevant constructs or phenomena for psychedelic-assisted therapy include: the ‘therapeutic alliance’—i.e., the presence of a collaborative relationship that includes agreement on treatment goals/tasks and a positive emotional bond (Bordin, 1979; Martin et al., 2000), and the psychoanalytic concept of “transference” or the unconscious ways that a person’s experience of past relationships influences their perception and experience of the therapeutic relationship and “countertransference” which refers to the reciprocal feelings evoked in the therapist (Horvath, 2000; Lemma, 2015). Contemporary thinking acknowledges the complexity of trying to theoretically define the therapeutic relationship as it is intersubjective and dynamic, influenced consciously and unconsciously by both therapist and patient (Horvath, 2000; Safran et al., 2001; Lemma, 2015). Compelling evidence exists that the quality of the therapeutic alliance is an important predictor of treatment outcomes in psychotherapy (for a review, see Baier et al., 2020). The therapeutic alliance is also recognised to be a dynamic phenomenon that is sensitive to change over the course of therapy; with a bidirectional and reciprocal relationship with symptom improvement; and which is influenced by pre-treatment patient characteristics e.g., complex relational trauma (Falkenström et al., 2013; Baier et al., 2020; Flückiger et al., 2020); and therapist characteristics and qualities (Ackerman and Hilsenroth, 2003; Phelps, 2017). The term “therapeutic rapport” is sometimes used synonymously with “therapeutic alliance”, albeit with a more specific stress on the *positive* nature of the emotional bond between the therapist and patient (Leach, 2005).

Despite strong theoretical assumptions surrounding the importance of the therapeutic relationship within psychedelic-assisted therapy, and strong evidence for its importance within psychotherapeutic interventions broadly, little empirical research has been done to examine its role in shaping psychedelic experiences and psychedelic-assisted therapy treatment outcomes. One prospective, observational survey study looking at people taking psychedelics in non-clinical settings found that having a “good feeling” or a “good relationship” with the people that an individual was taking a psychedelic with, or who would be taking care of them during the experience, was predictive of a reduced likelihood of challenging experiences and greater subsequent improvements in psychological well-being (Haijen et al., 2018). A similarly designed study of psychedelic use in communal settings, e.g., retreat centres, found that the positive relationships between rapport and increases in well-being and social connectedness, were mediated by experiences of togetherness and shared humanity (Kettner et al., 2021).

Although many psychedelic therapists have published qualitative reports of intensified transference in psychedelic-assisted therapy (Walsh and Grob, 2005; Grof, 2008; Fisher 2015), there has been no published quantitative research into the matter of more concrete questions, such as whether the quality of the therapeutic relationship is predictive of the quality of the acute psychedelic experience (e.g., mystical-type or emotional breakthrough experiences) en route to positive treatment outcomes. It is also important to consider what impact a weaker therapeutic alliance has on the psychedelic experience and clinical outcomes, as this may have implications for screening and therapeutic approaches e.g., considering who and at what point in a therapeutic process participants will benefit from the experiences, identifying those at risk of harm and to consider how the therapeutic framework might need to adapt in certain cases. Furthermore, research has not yet examined whether the strength of the therapeutic relationship leads to treatment outcomes *through its influence* on acute psychedelic experiences. Additionally, despite evidence for the dynamic nature of the therapeutic relationship and suggestions that psychedelic experiences and psychedelics themselves may intensify and potentially enhance the therapeutic relationship (e.g., Grinspoon and Bakalar, 1986; Grinspoon and Doblin, 2001; Walsh and Grob, 2005; Grof, 2008; Fisher 2015; Dolder et al., 2016), research has not yet examined whether psychedelic experiences are associated with subsequent improvements in the therapeutic relationship.

The present article uses data from a recently published DB-RCT (Carhart-Harris et al., 2021a) in which individuals with MDD received two treatment sessions of psilocybin (25 mg) plus 6 weeks of daily placebo; or two treatment sessions with a *de facto* placebo, plus 6 weeks of daily escitalopram. Both arms received therapeutic support as described below (see Methods). Due to our strong prior hypotheses in relation to the role of therapeutic alliance and rapport in mediating the quality of the psychedelic experience and subsequent key clinical outcomes, we chose to focus specifically on the psilocybin condition, but analysis of the escitalopram arm is available in the supplementary material. Our specific main aims were to:

**1**: Examine whether the quality of the acute experience (specifically, mystical-type experiences and emotional breakthrough) serves as a mediator of the relationship between the strength of the therapeutic relationship (specifically, therapeutic alliance measured here using the participant version of Scale To Assess the Therapeutic Relationship (STAR-P) and rapport which was measured via a single, self-constructed visual analogue scale) and changes in depression.

**2:** Explore the relationship between the strength of the therapeutic relationship (therapeutic alliance and rapport), the acute experience (mystical-type experience and emotional breakthrough) and changes in depression score (measured via the 16 item self-rated Quick Inventory of Depressive Symptomatology or ‘QIDS’) and the interplay between these factors on a session-by-session basis, given there were two psilocybin dosing sessions in this trial.

## Methods

### Trial Oversight

This study received approval from the Brent Research Ethics Committee (REC), the Medicines and Healthcare products Regulatory Agency (MHRA), the Health Research Authority (HRA), and the Imperial Joint Research Compliance (study sponsors) and General Data Protection Regulation offices. It also received a study-specific Schedule I license approval from the United Kingdom Home Office to prescribe psilocybin. All clinical visits took place at the National Institute for Health Research (NIHR)-funded Imperial Clinical Research Facility. The psilocybin for this trial was provided by COMPASS Pathways and the placebo tablets by the Pharmacy Manufacturing Unit at Guy’s and St. Thomas’ Hospital.

### Participants

**FIGURE 1:**
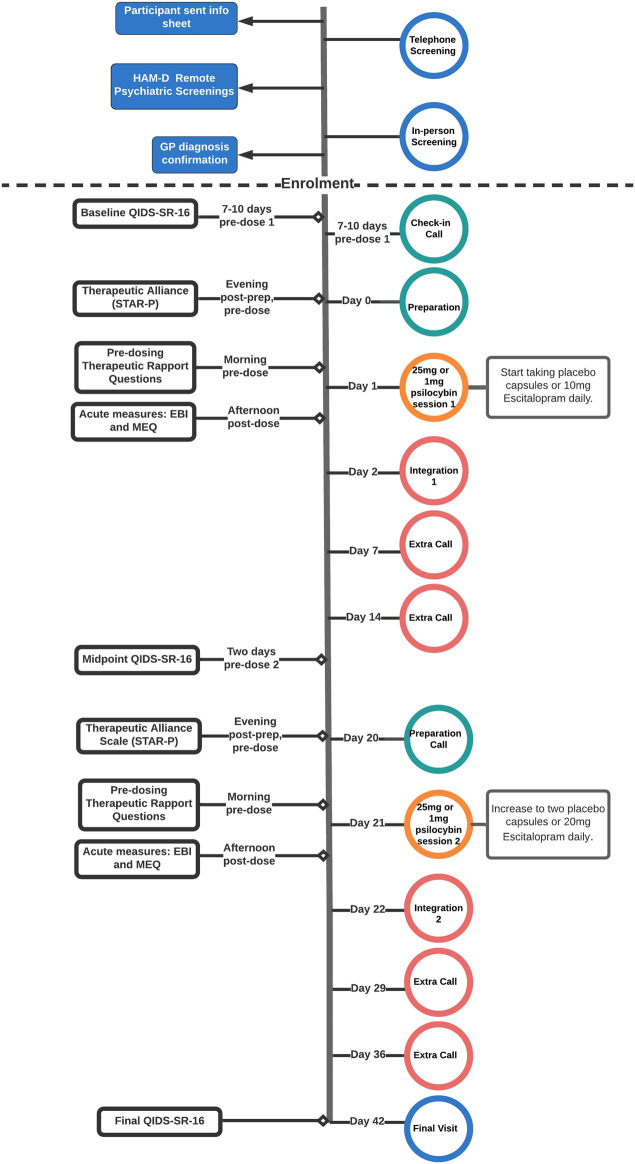
Flow-chart outlining study procedures for the psilocybin and escitalopram arms of the trial featured in this article, outlined in “Design and Procedures”. All participants included in this analysis followed the outlined procedures, unless otherwise stated in the “Descriptives” section of the results. After the 6-weeks key endpoint, participants completed remote monthly follow-up assessments until 6 months post-study. The dotted line separates pre-enrolment screening procedures (above line) from post-enrolment study procedures (below line). Info Sheet: Participant Information Sheet, HAM-D: 17-item Hamilton Depression Scale (clinician-rated), GP: General Practitioner, STAR-P: Scale To Assess the Therapeutic Relationship (participant-rated version), QIDS-SR-16: 16-item Self-Reported Quick Inventory of Depressive Symptomatology (primary outcome), EBI: Emotional Breakthrough Inventory, MEQ: Mystical Experience Questionnaire.

Detailed information about recruitment and the screening process including full exclusion criteria can be found in the supplementary material and clinical protocol published in Carhart-Harris et al. (2021a). The following summary is provided for ease of reference (see Figure 1 for a visual summary), however:

This study was advertised to General Practitioners via the NIHR Clinical Research Network and to the public online, recruiting men and women aged 18–65 years old. Telephone screening was followed by a remote Hamilton Depression (HAM-D; Hamilton, 1960) assessment with a minimum score of 17, indicating moderate-to-severe MDD, was required in order to proceed. Exclusion criteria included an immediate family or personal history of psychosis, major physical health conditions, a history of high risk suicide attempts, pregnancy, contraindications to selective-serotonin reuptake inhibitors (SSRIs) or magnetic resonance imaging (MRI), having failed a previous full course of escitalopram or having already taken psilocybin for therapeutic purposes.

Ahead of potential enrolment, the final stage was a face-to-face screening visit involving physical and psychiatric assessments with the study clinicians. After this, the participants met with their guiding pair for an hour. Discussion topics were typical of a psychotherapy initial assessment including current difficulties, salient early life experiences, family and interpersonal history, previous traumas and experiences of therapy. Participants had only two face-to-face contacts and one phone call with their therapists prior to dosing, thus, an ability to build sufficient therapeutic alliance and rapport relatively quickly was deemed necessary for inclusion. This was assessed by the team clinically as in traditional therapy assessments based on how they related during the screening process e.g. their ability to speak openly about their difficulties; to be emotionally vulnerable; an awareness of and ability to speak about their own relational patterns and how this might impact on the therapeutic relationship. Information around current and historical relationships and previous experiences of therapy was also gathered. More concretely those with a suspected or known diagnosis of any conditions which would significantly impact on rapport such as emotionally unstable personality disorder or complex post-traumatic stress disorder (as assessed by the screening psychiatrist) were excluded.

All participants withdrew from psychiatric medication for at least 2 weeks before starting the trial (Table 1 for detailed demographics) and psychotherapy for at least 4 weeks.

**TABLE 1:** Demographic information of participants in the psilocybin arm of the trial.

Overall	
Total patient number included in model	29
Total patient number excluded from model	1
**Demographics of those included**	
Age, years—mean, SD (range)	42.8, 11.6 (21–64)
Females, number (%)	11 (38)
Caucasian Ethnicity, number (%)^a^	27 (93)
Employment status—number (%)	
Employed	20 (69)
Unemployed	7 (24)
Student	2 (7)
University-level education, number (%)	22 (76)
Past psilocybin use, number (%)	7 (24)
Weekly alcohol (United Kingdom units)—mean, SD (range)	4.8, 5.4 (0–20)
**Clinical**	—
Illness duration, years—mean, SD (range)	22.2, 10.9 (3–44)
HAMD-17 scores at pre-treatment baseline-mean, SD (range)^b^	19.2, 2.3 (16–23)
QIDS-16 scores at pre-treatment baseline—mean, SD (range)^c^	14.7, 3.9 (7–23)
No. past psychiatric medications—mean, SD (range)	2.2, 1.7 (0–6)
Discontinued psychiatric medication for trial, number (%)	11 (38)
Past psychotherapy, number (%)	27 (93)

Pre-treatment baseline was 7–10 days before dosing-day 1.

a Race was reported by the patients.

b The scores on the 17-item Hamilton Depression Rating Scale (HAM-D-17) range from 0 to 50, with higher scores indicating Greater depression. At screening, which was typically a few weeks before pre-treatment baseline, all the patients. Had a score of at least 17 on the HAM-D-17. The depression scores reported in this table are from pre-treatment baseline. And not screening.

c The scores on the 16-item Quick Inventory of Depressive Symptomatology–Self-Report (QIDS-SR-16) range from 0 to 27, with higher scores indicating greater depression.

### Design and Procedures

This was a phase II, double-blind, two-armed, randomised controlled trial. In this study, participants with moderate-severe MDD were randomised either to an ‘escitalopram arm’ or to a ‘psilocybin arm’. The analyses in this paper focus primarily only on the latter arm, but full trial procedures and main clinical results for both arms have been reported in Carhart-Harris et al. (2021a). In order to validate our findings, we ran the same models used in our primary confirmatory analysis for the escitalopram arm. This data is available in the supplementary material of this paper.

Each participant was assigned two guides (see introduction). These were mostly trained or training clinical psychologists, psychotherapists or psychiatrists with therapy experience. All guides had experience of working with psychedelics or non-ordinary states. One of the pair was assigned the role of ‘main guide’ and was present in all six of their face-to-face visits and five calls post-screening (plus the option of four additional if clinically indicated). One of the two guides could be a clinical trainee if accompanied by an experienced mental health professional.

See Figure 1 for a visual summary of all clinical contacts. Approximately 1 week (seven to 10days) prior to the first psilocybin dosing session guides rang participants (Check-in Call) to discuss practicalities, answer questions and to discuss the music playlist as participants were given the option to add three personal music choices to play at the end of the session. In visit 1 (“Prep”) all participants were given approximately 5 hours of preparation by their guides for the next day’s psilocybin session. In visit 2 (Dosing Day 1), participants in the psilocybin arm were given 25 mg of psilocybin and participants in the escitalopram were given 1 mg of psilocybin, accompanied and supported by their two guides throughout the day. All participants were told that they would definitely be given psilocybin, but they were not told the doses in an effort to standardise expectations. After psilocybin administration, the guides provided the participant with psychological support throughout the day. The acute effect of a 25 mg psilocybin dose lasts approximately 6 hours. The morning after dosing, participants returned for an ‘integration’ session (visit 3), where they discussed their psilocybin experience with their guides for two to 3 hours. At the end of the dosing day, patients on the psilocybin arm were given a 3-week course of placebo capsules and the escitalopram arm were given capsules containing 10 mg of escitalopram. All patients were instructed to take them every morning for the duration of the trial. Patients had the option of weekly 1-h integration or therapy calls with their main guides for the duration of the trial, as clinically indicated.

Three weeks after the first psilocybin session, patients had a second 25 mg (psilocybin arm) or 1 mg (escitalopram arm) psilocybin dosing day (visit 4) and a next day integration session for two to 3 hours (visit 5), preceded by a shorter preparation call over the phone. Patients on both sides were instructed to start taking two tablets every day, increasing the escitalopram dose to 20 mg for the escitalopram arm.

Three weeks after the second integration session, patients returned for a 3-h final visit [visit six; final follow-up (FFU)]. This was their final formal integration with their guides within the 6-weeks trial, where they could discuss their experience of the trial and their current mood state. After performing key assessments, patients and clinicians were unblinded at this key endpoint so that the clinical team and patients could consider future treatment options and support.

### Therapy Protocol

Detailed information about the role of the guides and the therapy protocol can be found in the supplementary material of Carhart-Harris et al. (2021a). See (Watts and Luoma, 2020) for the original publication of the Accept-Connect-Embody (ACE) therapy model, and (Watts, 2021) for an associated manual.

In keeping with standard practice in psychedelic-assisted therapy (Grinspoon and Doblin, 2001; Grof, 2008; Johnson et al., 2008; Richards, 2016; Nayak and Johnson, 2020), the therapeutic protocol used in this trial consisted of preparation, support during psilocybin sessions, and integration therapy. Principles of Acceptance and Commitment Therapy and psychological flexibility (Hayes et al., 2012), which are being increasingly utilised in the psychedelic therapy field (Walsh and Thiessen, 2018; Luoma et al., 2019; Sloshower et al., 2020), provided a particular source of inspiration for the therapy team, where the latter construct, in particular, has been shown to be an important mediator of therapeutic outcomes in psychedelic-assisted therapy (Close et al., 2020; Davis et al., 2020; Zeifman et al., 2020). A psychedelic-specific therapy model, informed, in part, by ACT, emerged during the trial, and is referred to as The ACE model (Watts and Luoma, 2020). The ACE model emphasises building trust between participants and their guides, engaging with and accepting difficult emotions (in contrast to experiential avoidance); connecting to personal meaning, values, self and others; and giving mindful awareness to embodied or somatic experiences (Watts and Luoma, 2020). Guides were encouraged to support participants in a non-directive and open manner, showing unconditional positive regard throughout (Rogers, 1951).

A primary aim of the preparation session was building trust and rapport, setting intentions and sharing information about psychedelic experiences. Emphasis was placed on ideas of moving towards difficult experiences and painful content. Prior consideration was given to how the guides would support the participant during the sessions. The participants feelings in relation to asking for help were explored. Discussions were had around physical contact, such as the use of hand holding to provide non-verbal grounding and support during the experience. Visualisation exercises were used as a preparation and integration tool to support processes of attuning to their bodies, moving towards difficult emotions, and connecting to self and others, details of which can be found in the original publication on the ACE model (Watts and Luoma, 2020; Watts et al., 2021).

For the psilocybin sessions themselves, participants wore eye masks and earphones through which they listened to a pre-selected playlist. This playlist was informed by research demonstrating the influence of music, i.e. “the hidden therapist”, on the subjective experience and clinical outcomes (Kaelen et al., 2018). Therapists aimed to provide a safe and containing setting with a view to allowing unconscious material, emotions, memories and difficulties to emerge. Participants were encouraged to direct their attention inwards and share their experiences towards the end of the day, if they wished. Integration sessions took place in person the following day and in subsequent weekly calls if indicated. Integration sessions involved open and attentive listening to participants’ accounts of their experiences and reflections. The role of the guide during integration is to provide compassionate support and recognition of any psychological insights or changes.

Monthly group supervision was provided by a consultant psychiatrist and psychotherapist, with extensive experience of working with non-ordinary states.

### Measures

**Participant Demographics and Histories** *—*Age, gender, ethnicity, education, employment, and clinical history including illness duration, past psychotherapeutic and pharmacological treatments were collected at screening.

**Primary Outcome** *—*The 16 item self-rated Quick Inventory of Depressive Symptomatology (QIDS; Rush et al., 2003) was used to assess changes in depression severity (range of scores = 0–27). The QIDS was administered at three timepoints: 1) baseline, seven to 10 days ahead of the first psilocybin session; 2) mid-treatment, 3 weeks after the first dosing and 2 days ahead of the second psilocybin session; 3) post-treatment/primary endpoint, 3 weeks after the second psilocybin session.

**Therapeutic Relationship** *—*The participant-rated Scale To Assess the Therapeutic Relationship (STAR-P; McGuire-Snieckus et al., 2007) was used to assess the therapeutic alliance (specifically the constructs of positive collaboration, positive clinician input and non-supportive clinician input) 1-day pre-dose one and 1-day pre-dose two. A self-constructed single-item measure of rapport (“*I have a good relationship with the main person/people who will look after me during the upcoming experience”*) rated on a 0–100 Visual Analogue Scale (VAS) assessed the extent of rapport in the hours immediately before the psilocybin session, part of the Psychedelic Predictor Scale (Haijen et al., 2018).

**Acute Experience Measures** *—*On the afternoon of each dosing day, after psychedelic effects had worn off, participants completed the Mystical Experience Questionnaire (MEQ; Barrett et al., 2015), which assesses mystical-type peak experiences using 6-point Likert scale, as well as the Emotional Breakthrough Inventory (EBI; Roseman et al., 2019), a 6-item (0–100 VAS) measure of cathartic release and resolution of difficult emotions or trauma.

### Statistical Analyses

**Primary Confirmatory Analysis** *—*Longitudinal path modelling was employed to test the primary hypothesis, that therapeutic alliance would affect treatment outcomes by modulating the intensity of acute subjective effects. Based on previous research (Roseman et al., 2018; Roseman et al., 2019), mystical-type (MEQ) and emotional breakthrough experiences (EBI) were chosen as acute predictors of depression outcomes. Therapeutic alliance was measured on the day before each psilocybin session using the STAR-P. The single-item participant-rated measure of their felt rapport with their guides was taken within 3 hours prior to dosing. These values were included in the path model to account for variation in therapeutic rapport experienced by participants on the day of the session.

In order to simplify the analysis across the two separate psilocybin sessions while still taking into account all available data, the respective maximum MEQ and EBI score across the two sessions was used to predict depression outcomes. This was done based on the hypothesis that the predictive function of subjective effects on outcomes would depend on the single most intense experience across the two sessions (rather than accumulative). Due to expected multicollinearity between the EBI and MEQ (*r* = 0.61 across both sessions), separate models were fitted including maximum EBI and MEQ scores, respectively, as well as the relevant STAR-P and Rapport scores (i.e., from the same respective dosing day on which the higher EBI or MEQ score was reported). Models with the same structure (using maximum EBI and MEQ) were constructed and analysed including subjects in the escitalopram arm, which are reported in the supplementary material.

**FIGURE 2:**
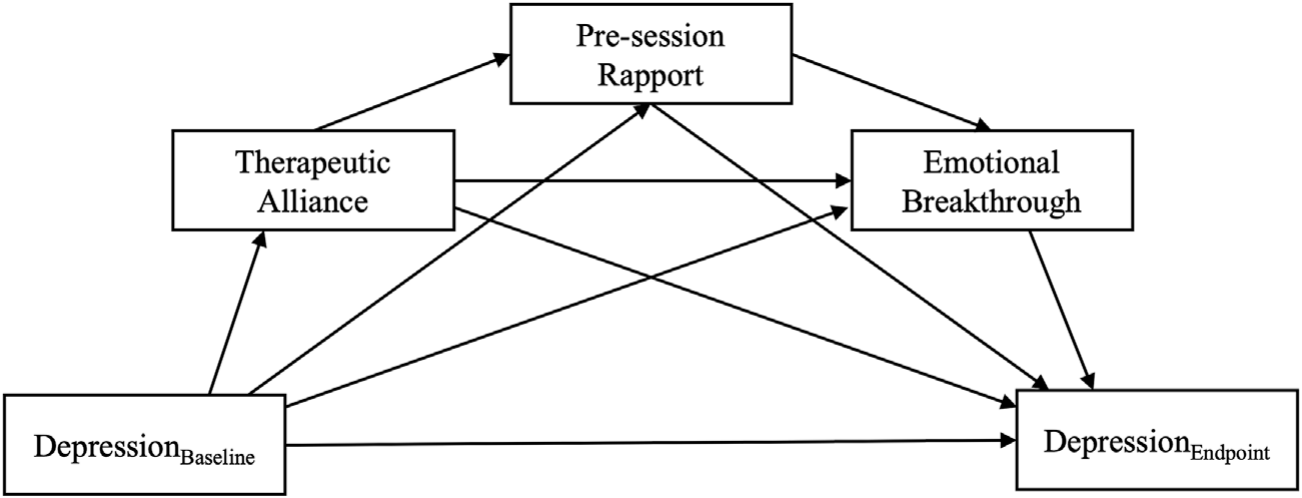
Directed Acyclic Graph (DAG) displaying the path structure of a fully saturated sequential mediation model testing the serial mediation effect of therapeutic alliance on depression outcomes via the active psychedelic experience. The model is completely unrestricted, meaning that all possible paths between all variables are specified.

**Secondary Exploratory Analyses** *—*In order to investigate session-specific mechanisms of therapeutic alliance and the acute psychedelic experience, additional (near-) saturated sequential mediation analyses were conducted for the first and second psilocybin session, a special case of path model in which all possible paths between all variables are specified. Saturated models by definition reproduce the data perfectly but are also more susceptible to random noise in the data. The specification of all possible paths between variables makes this type of model especially useful for exploratory analyses that are not hypothesis driven. For both the first and second session, covariates included therapeutic alliance, rapport, MEQ and EBI (the latter two in separate models to mitigate multicollinearity issues). Outcomes in both sessions included depression severity at the key endpoint, corrected for baseline depression severity in case of the first, and intermediate depression severity in case of the second psilocybin session. This was done in order to investigate to what extent the subjective effects during the first and second session would independently contribute to overall changes in depression severity. As an additional outcome in the first psilocybin session, therapeutic alliance ahead of the second session was included to assess whether participant experience during the first psilocybin session would impact the therapeutic relationship and thereby indirectly affect the second psilocybin session. Additional paths between baseline (or in the case of session two, intermittent) QIDS scores and other included covariates led to (near-) saturation of the models, taking a general shape as displayed in Figure 2.

For all path analyses, maximum likelihood estimation was chosen as the estimation method, since all endogenous variables were continuous. Following the recommendation by Kline (2015), multiple indicators of model fit are reported for confirmatory analyses, including model Chi-Square, Comparative Fit Index (CFI) and Root Mean Square Error of Approximation (RMSEA). Simulation studies have shown that small sample sizes lead to an overestimation of fit for Chi-square, and an underestimation of fit for CFI and RMSEA (Shi et al., 2019), the latter thus being more conservative indicators in the present study. Thresholds for acceptable and good fit were taken from the extant literature, where less stringent cut-offs were chosen for RMSEA to account for sample size biases (Hooper et al., 2008; Hair et al., 2010; Awang, 2014). Effect strengths of standardized regression coefficients were interpreted following Acock and Oregon 2018), β < 0.2 being weak, 0.2 < β < 0.5 moderate, and β > 0.5 a strong effect.

While the trial, including primary and secondary outcomes published in Carhart-Harris et al. (2021a) was registered at Clinicaltrials.gov, 2021 (Identifier NCT03429075), the specific analyses presented here had not been pre-registered.

## Results

### Demographic Information

Approximately, 1,000 individuals were screened by telephone and/or email. This number is large due to the large volume of interest in the trial, due to high public awareness of our psychedelic research programme and demand for psilocybin-therapy in particular. 891 did not meet inclusion criteria and 50 chose not to participate. A total of 103 individuals were invited to an in-person screening visit. At the point of face-to-face screening, 44 participants were excluded because of potential difficulties in building alliance and rapport. Finally, 59 participants with moderate-severe major depressive disorder were enrolled onto this trial. They were randomised to one of two groups (see Procedures section): 30 into the psilocybin arm and 29 into the escitalopram arm. As the analyses in this paper focus solely on the psilocybin arm, demographics for this sub-group are provided in Table 1 below and demographics for the escitalopram arm is available in the supplementary material. One participant has been excluded from this analysis because they did not comply with required restrictions on drug-taking throughout the trial which was felt to impact on the therapeutic relationship, acute experience and outcomes. Other protocol deviations include two participants who were unable to complete their second psilocybin dosing and integration visits due to Covid-19-related restrictions, and one participant who stopped taking their placebo tablets after guessing their content. These individuals have, however, been included in this paper’s analyses as the nature of their deviations from the protocol does not corrupt or invalidate their data meaningfully.

### Primary Confirmatory Analysis: Therapeutic Alliance Predicts Outcomes via Acute Subjective Effects

**FIGURE 3:**
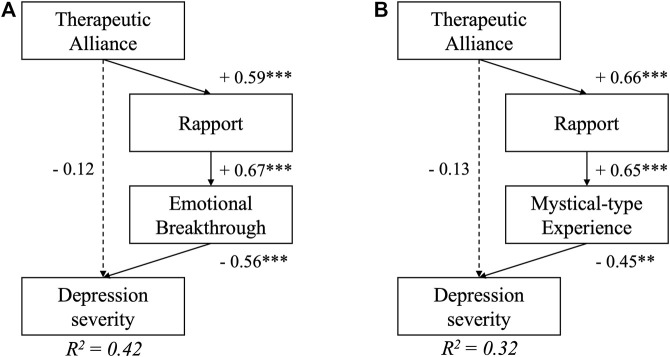
Path models testing the primary hypothesis that therapeutic alliance would lead to better depression scores 6 weeks following psychedelic-assisted psychotherapy. Serial mediation of therapeutic alliance via pre-session rapport and A) Emotional Breakthrough and B) Mystical-type experiences were supported by the models. Depression severity at the 6-weeks Endpoint was adjusted for baseline depression scores (*p* > 0.1, not displayed in the figure), which by itself accounts for *R* ^2^ = 0.12, i.e., 12% of variance in the final outcome. Numbers represent standardised regression coefficients for significant (solid) and non-significant (dashed) paths. **indicates *p* < 0.01, *** *p* < 0.001.

As displayed in Figure 3, depression severity at the key endpoint was significantly predicted by both EBI and MEQ scores, but the standardised effects were slightly larger for EBI (β = −0.56, *p* = 0.03) as compared with MEQ (β = −0.45, *p* = 0.04) scores, suggesting that emotional breakthrough was a more reliable predictor of depression improvements in this trial, than MEQ assessed “mystical-type experience”. This difference was also reflected in a higher amount of explained variance in the final depression severity outcome in a model that included the EBI, vs one that replaced it with the MEQ: *R* ^2^ = 0.42 vs 0.32, respectively. After subtracting the amount of variance explained by baseline depression severity alone, the EBI-based model explained 42–12% = 30% and the MEQ-based model explained 32–12% = 20% of variance in depression severity at the key endpoint. In both cases, the cumulative indirect effect of STAR-P on depression outcome was significant (β = −0.22, *p* = 0.02 for EBI-based model; β = −0.19, *p* = 0.03 for the MEQ-based model), although this effect would have been reduced to trend level (*p* = 0.06) in case of the MEQ after applying Bonferroni correction for the two comparisons. Both models had acceptable to good fit on all estimated fit indices (see Table 3 for fit estimates of all included path models).

### Secondary Exploratory Analyses: Psilocybin Session-specific Effects

**FIGURE 4:**
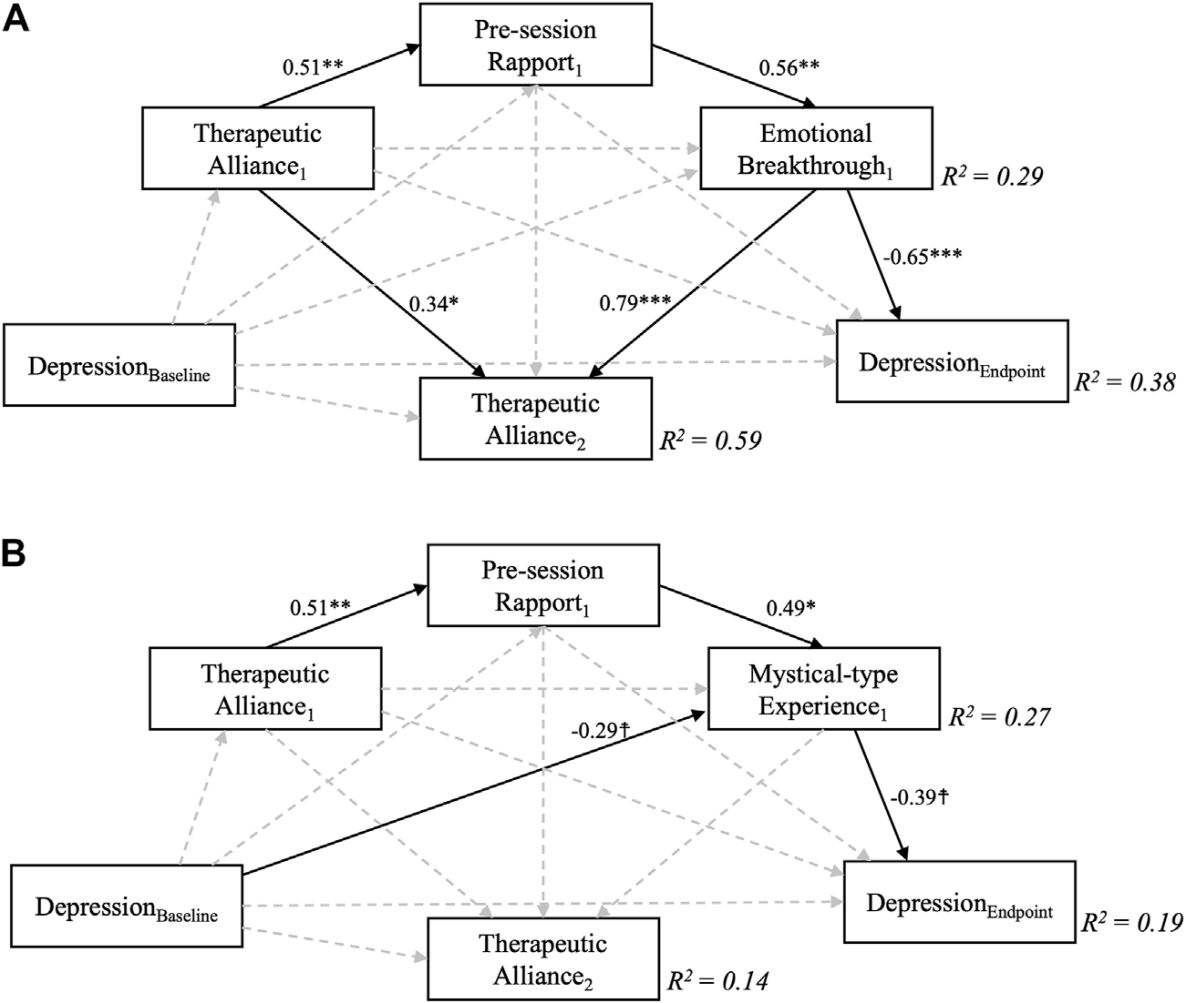
Near-saturated sequential mediation models exploring the relationship between therapeutic alliance during the first psilocybin session and depression scores 6 weeks following a two-dose course of psychedelic-assisted psychotherapy. Sequential mediation of therapeutic alliance via pre-session rapport and A) Emotional Breakthrough and B) Mystical-type Experiences were supported by the models in the case of depression severity, but not for intermediate therapeutic alliance measured ahead of the second psilocybin session, which was only significantly predicted by Emotional Breakthrough scores. Depression severity at 6 weeks was controlled for baseline depression scores, which by itself accounted for *R* ^2^ = 0.12, i.e., 12% of variance in the final outcome. MEQ, but not EBI scores were furthermore affected by baseline depression severity, although only at trend level. Numbers represent standardised regression coefficients for significant (solid, *p* > 0.1) but not non-significant (dashed) paths. Subscript numbers refer to the different psilocybin sessions, one and two; e.g., Therapeutic Alliance_1_ refers to STAR-P scores ahead of psilocybin session one and Therapeutic Alliance_2_ refers to STAR-P scores ahead of psilocybin session 2. ☨ indicates *p* < 0.1, *p < 0.05, **p < 0.01, ***p < 0.001.

Results for the near-saturated mediation models for the first psilocybin session are displayed in Figure 4. The EBI (*β* = −0.65, *p* < 0.001) significantly mediated the effect of therapeutic alliance on depression severity but the same was not quite true for the MEQ (*β* = −0.39, *p* = 0.06). Therapeutic alliance ahead of the second psilocybin session was significantly affected only by EBI (*β* = 0.79, *p* < 0.001), but not MEQ scores (*β* = −0.02, *p* = 0.94). Thus, while 59% of variance in STAR-P scores was explained in the EBI model, the MEQ-based model only explained 14% of variance in STAR-P scores, which approximately corresponds to the amount of variance accounted for by baseline STAR-P scores. A trend-level relationship was found between baseline QIDS scores and MEQ scores, meaning that higher baseline depression severity was associated with lower mystical-type experience scores for the first psilocybin session (*β* = −0.29, *p* = 0.09).

**FIGURE 5:**
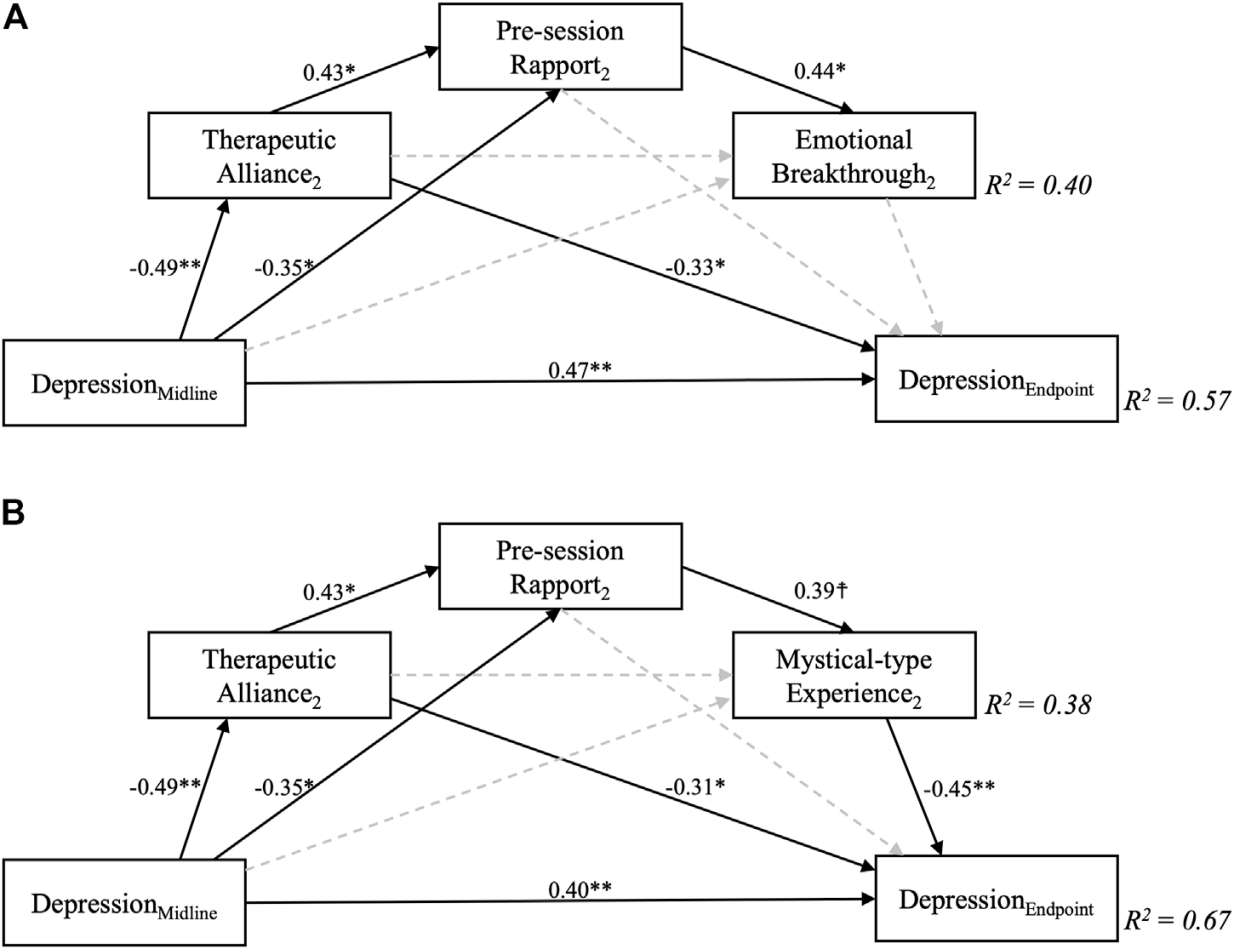
Fully saturated sequential mediation models exploring the relationship between therapeutic alliance during the second psilocybin session and depression scores 6 weeks following a two-dose course of psychedelic-assisted psychotherapy. Sequential mediation of therapeutic alliance via pre-session rapport and B) Mystical-type Experiences, but not A) Emotional Breakthrough was supported by the models for this second session. Importantly, depression severity at 6 weeks was controlled for midline (3 weeks post-first dose) depression scores, which by itself accounted for *R* ^2^ = 0.55, i.e., 55% of variance in the final outcome. Greater midline depression severity significantly predicted worse therapeutic alliance and pre-session rapport scores. Decimal values represent standardised regression coefficients (β values) for significant (solid, *p* > 0.1) but not non-significant (dashed) paths. The subscript number two refers to the second psilocybin session. ☨ indicates *p* < 0.1, *p < 0.05, **p < 0.01, ***p < 0.001

Results for the saturated mediation models for the second session are displayed in Figure 5. When controlling for intermediate QIDS scores, only MEQ (*β* = −0.45, *p* = 0.002) but not EBI (*β* = −0.18, *p* = 0.27) scores significantly affected final depression outcomes. Instead, significant direct effects between therapeutic alliance and depression outcomes emerged for each model (*β* = −0.33, *p* = 0.049; *β* = −0.31, *p* = 0.033, for MEQ and EBI-based models, respectively). A large overall amount of variance in final depression outcomes was explained in each of the fully-saturated mediation model (*R* ^2^ = 0.67, and 0.57), but it is important to note that the correlation between depression severity at the intermediate and the final endpoints was very high at *r* = 0.74, thus already accounting for 0.74^2^ = 55% of variance. The implication is that most of the mediation occurs in relation to the first dosing session, after which depression severity prior to session two is a strong predictor of depression severity at the 6-weeks endpoint. The additional amount of variance in final depression severity outcomes explained by inclusion of other covariates than midline depression was thus only approximately 67–55% = 12% for the MEQ-based model, while the inclusion of EBI scores and other covariates during the second session only explained 65–55 = 2% of additional variance. Of interest, significant negative effects between midline QIDS and therapeutic alliance (*β* = −0.49, *p* = 0.004), as well as pre-session rapport (*β* = −0.49, *p* = 0.036) were revealed - both in the EBI- and MEQ specific models, meaning that higher depression severity at the intermediate (3 weeks post-session 1) timepoint were associated with worse therapeutic alliance and rapport ahead of the second session. Again, this finding could be viewed as highlighting the importance of processes leading up to and including dosing session one as being the major determinants of eventual outcomes at the study endpoint.

### Supplementary Result

See supplementary material for the path models for the escitalopram arm (Supplementary Figure S1), the histograms comparing the acute scores between the psilocybin and escitalopram arm, i.e., EBI and MEQ for dosing one and two (Supplementary Figure S1), the demographics of this arm (Supplementary Table S1), Pearson correlations between all measures that included in the path models (Supplementary Table S2) and a correlation matrix of all included variables on the psilocybin arm (Supplementary Table S3).

## Discussion

The importance of the therapeutic relationship in psychedelic-assisted therapy has long been treated as a matter of conventional wisdom, both traditionally and in contemporary research (Grof, 2008; Johnson et al., 2008; Richards, 2016; Carhart-Harris and Nutt, 2017; Breeksema et al., 2020; Nayak and Johnson, 2020; Watts & Luoma, 2020) but has never been empirically tested. Here, via a sequential path model, we have demonstrated how a strong therapeutic alliance predicts pre-session rapport, which predicts greater emotional breakthrough and “mystical type” experience towards improved clinical outcomes in a trial of psilocybin-assisted therapy for depression. This work shows how the acute experience, previously established as critical to outcomes (Watts et al., 2017; Roseman et al., 2018; Johnson et al., 2019), is influenced by pre-session therapeutic alliance and rapport, supporting the idea that participants need to feel safely contained within the therapeutic relationship in order to surrender to the psychedelic experience and the vulnerabilities it may open up.

As the field of psychedelic-assisted therapy develops, it is essential to test even the most confidently held assumptions, such as the importance of the therapeutic relationship, to optimise clinical outcomes and challenge economic or commercial imperatives that might lead to risky cutbacks in therapeutic support, as well as its supervision and training (Noorani 2020; Marseille et al., 2020; Carhart-Harris et al., 2021b). It is also important to consider how the nature and quantity of therapy might need to adapt to more complex clinical populations. Such individuals e.g., with greater personality and attachment difficulties, may struggle to build trust or to feel safe in relationships. No research to date has looked at using psychedelics in patients with emotionally unstable or borderline personality disorder (Zeifman and Wagner, 2020). As in many psychedelic clinical trials, a diagnosis of a personality disorder, complex trauma or an inability to build early alliance or rapport were exclusion criteria in this trial (Carhart-Haris et al., 2021a). Whether such complexity is a contraindication for psychedelic-therapy is an important question. If it is not, then how might therapeutic models adapt to accommodate and support these participants? These matters are important, particularly given the complexity of treatment-resistant populations, the treatment of which both MDMA and psilocybin trials have been exploring, where e.g., there are high rates of comorbid personality disorders and complex relational trauma (Thase, 1996; Carhart-Harris et al., 2021b).

The finding of our stage-by-stage analysis may bear relevance to a stage and relationship sensitive adaptive treatment approach going forwards. For example, in the first psilocybin session, emotional breakthrough appears to be the most potent predictor of subsequent improvements in depressive symptoms and this relationship is mediated by pre-session therapeutic alliance and rapport (see Figure 4A). The implications of this model are relatively straightforward therefore, merely highlighting the importance of the therapeutic alliance, in line with prior assumptions, but also placing a significant emphasis on the clinical importance of emotional release or catharsis in the first session.

A second major implication of the model results is that the picture changes somewhat ahead of the second psilocybin session, with emphasis placed less on emotional breakthrough and more on the mystical-type experience as a means to further therapeutic progress. Also, of note, the therapeutic alliance ahead of the second session has a direct impact on final depression scores, now in a manner that is *not* mediated by the acute experience. Perhaps the most interesting nuance in this regard is that if depression scores are high at this treatment midpoint, therapeutic alliance and rapport is weak—and this is critical, as weaker alliance at this key treatment mid-stage translates into poor depressive outcomes at the end of the trial, as well as lower levels of mystical-type experience and emotional breakthrough (Figure 5B) These findings have significant clinical implications, further discussed below.

### The Therapeutic Relationship

On a broader level, our results consolidate, but now in the context of psychedelic-assisted therapy, the view that having a strong therapeutic relationship shapes the quality of the therapeutic process en route to better clinical outcomes. An important caveat prefacing this discussion is the particular context of the present trial’s double-blind and randomised design, and the “promissory culture” surrounding psychedelic-assisted therapy at present. This can impact on scientific research in an unhelpful way, e.g., causing exaggerated expectations and an idealisation of researchers and guides. Inflated expectations can increase the risk of disappointment, as well as feelings of neglect or rejection if expectations are not met.

In the present study, the therapeutic alliance was either stronger (e.g., McGuire-Snieckus et al., 2007) or similar (e.g., Knittle et al., 2019) to that observed in previous clinical samples. More research is required to examine what factors determine the strength of therapeutic alliance and rapport. Some candidates include therapist experience, training and interpersonal disposition; the length and quality of the pre-dosing preparation work (e.g., early alliance and rapport building); and specific patient factors (Horvath, 2000; Grof, 2008). The latter might be assessed by looking at patterns of relating, such as attachment style or the influence of internalised early relational experiences on lifelong patterns of relating (i.e. quality of object relations; Goldman and Anderson, 2007; Horvath, 2000). A link between early relational experiences and depression is well established, with attachment difficulties suggested to partially mediate the relationship between early interpersonal trauma and depression (Fowler et al., 2013). Recent research examining the relationship between insecure attachment styles and responses to individual psilocybin therapy sessions with group preparation and integrative group therapy sessions (Stauffer et al., 2021), found that high attachment avoidance was associated with more intensely challenging psychedelic experiences, whereas high attachment anxiety was associated with higher levels of mystical experience in the psilocybin sessions. Furthermore, attachment anxiety was significantly reduced by the trial endpoint.

In the present study, it is worth noting that nearly half of the variance of both maximum EBI and MEQ was explained by baseline depression, therapeutic alliance and rapport (Figure 2). Looking at the exploratory analysis, the results suggest that a strong therapeutic alliance and rapport prior to the first psilocybin session resulted in more emotional breakthrough, as well as better alliance and rapport before the second session, and better eventual clinical outcomes. This finding suggests the clinical importance of having a strong pre-session therapeutic alliance and rapport in place to support a participant in accessing and working through previously unprocessed memories, emotions or traumas during this first psilocybin session.

Focusing next on the second session, therapeutic alliance after the first session had a direct impact on final outcome in our second dosing model. That is, as shown in the top-left to bottom right diagonal in Figure 5, post session one (pre session two) therapeutic alliance predicted final depressive symptom severity independent of the nature of the acute experience within the second psilocybin session. This may speak to an enhanced importance of the therapeutic relationship as the main vehicle of change after a first psychedelic-assisted therapy session, perhaps due to a sub-acute intensification of transference (Walsh and Grob 2005; Grof, 2008; Fisher, 2015). Some consideration could be given here to prolonging the gap between psilocybin sessions, so as to develop the therapeutic relationship, giving it priority ahead of another psilocybin therapy session.

Having a strong therapeutic alliance appears to support participants towards more powerful emotional breakthroughs and mystical experiences, but the experience of coming into contact with old traumas and working through difficult emotional material in the context of a containing and supportive therapeutic relationship may be a key component of the healing *process*. Our data revealed that emotional breakthrough after session one was a significant predictor of therapeutic alliance prior to session two, but the same was not true for mystical-type experiences linked to session one. One speculative explanation for this could be that emotional breakthroughs are more inter-personal or relational in content and nature; whereas mystical-type experiences are more transpersonal, solitary experiences which transcend the personal or relational.

Gaining insight into relational patterns and narrative building at an explicit verbal level is considered a fundamental aspect of many psychological therapies, but alongside this, the experience of the relationship with the therapist(s) or guides(s) themselves can result in changes in relational knowing that occur at an unconscious or procedural level (Lachmann and Beebe, 1996; Lemma, 2015). The latter process may be particularly relevant to psychedelic-assisted therapy, with early psychedelic-assisted therapy and more recent therapy models emphasising more embodied, non-verbal experiential processes (Grof, 2008; Watts and Luoma, 2020). Psychedelics can induce heightened affective and age-regressed states (Grinspoon and Doblin, 2001; Grof, 2008; Watts et al., 2017; Carhart-Harris and Friston, 2019) in which care can be experienced in an intensified way, potentially recapitulating the early care and attachment processes of infancy (Lachmann and Beebe, 1996; Grof, 2008; Watts and Luoma, 2020). The neuroplastic brain state associated with psychedelics (Carhart-Harris and Friston, 2019; Murphy-Beiner and Soar, 2020; Olson, 2021) may serve to catalyse deep processes of change, including changes in implicit relational being and knowing, which may, in turn, support further therapeutic progress.

In a study where as many as 40% of participants in either trial arm failed to achieve remission and 30% failed to respond to psilocybin-assisted therapy, it is important to acknowledge that one implication of our results is that participants who reported *weaker* therapeutic alliance and rapport experienced *less* therapeutic breakthrough during sessions which appear necessary for improvement in core symptomatology by the trial end-point. One possible interpretation of this study’s results is that early warning signs of poor response, such as poor initial therapeutic alliance, perhaps combined with a lack of emotional breakthrough in a participant’s first psilocybin session, might signal a postponement of any subsequent psilocybin sessions. At such a juncture, the therapeutic focus might move towards working on aspects of the therapeutic relationship or trying to understand other unconscious factors that may be maintaining the illness - with a view to improving alliance and/or enhancing the likelihood of an emotional breakthrough. Arguably, the earlier clinicians can intervene in an adaptive, evidence-based way, the better. More concretely, this could imply assessing baseline predictors of poor therapeutic alliance.

Here we have shown that poor therapeutic alliance led to less emotional breakthrough and mystical experience during the psilocybin sessions. One might consider how specific patient factors might underlie both the quality of the therapeutic relationship and the psychedelic experience itself. What might look like a participant resisting the experience or not responding to psychedelics might just be a different kind of psychedelic experience. Further research is needed to better understand the quality of the psychedelic experience in cases of a weaker therapeutic alliance, e.g., is it associated with more challenging experiences (Stauffer et al., 2021), amplified psychological defences or intensified transference (Grof, 2008)? Moreover, how might these experiences be best supported and worked with therapeutically? Participants who experience more challenging therapeutic processes may need a different therapeutic approach, which could entail e.g., a longer preparation period to build trust and safety, greater number of psilocybin-therapy sessions and/or more integration therapy (Carhart-Harris et al., 2021b). There is a precedent of endeavouring to work with more challenging experiences e.g. amplified negative transference (relational difficulties); states of intensified shame, hopelessness, despair; in psychedelic-therapy dosing sessions (Grof, 2008), as there is a long tradition of in traditional psychological therapies (Piper et al., 2004; Yakeley, 2014; Lemma, 2015). However, this may be more challenging in the context of tightly controlled and constrained short-term clinical trials; compared to real-world clinical care contexts, permissive of greater flexibility, adaptability and longer-term care (Grof, 2008). Future studies may consider alternative clinical trial designs and therapeutic approaches that are more responsive and adaptive, potentially allowing for longer gaps between dosing sessions and more flexibility on dosage amount, regularity (Carhart-Harris et al., 2021b) and perhaps even drug—where e.g., MDMA-therapy could be explored as a switch option if clinically indicated (Mitchell et al., 2021).

### The Acute Experience

Our results demonstrate that depression severity scores following psychedelic-assisted therapy were significantly reduced in relation to the intensity of both emotional breakthrough and mystical type experiences. Greater standardised effects were seen for maximum EBI, reflected in a higher amount of variance in depression severity explained by mediation models including the EBI (see Figure 2). The predictive value of the acute experience on clinical outcomes demonstrated here is in line with previous research (for a review, see Romeo et al., 2021). This finding enriches recent debates about the necessity of the subjective psychedelic experience for enduring therapeutic effects (Olson, 2021; Yaden and Griffiths, 2021) and the increasing interest in developing non-hallucinogenic analogues of psychedelic compounds (Cameron et al., 2021).

Emotional breakthrough is arguably a more intuitive and familiar construct as a therapeutic mechanism, which may be more easily integrated into mainstream neuroscience and mental health care. Whilst recent advocacy for the secularisation of psychedelic treatment spaces (Johnson, 2021) is a welcome call to prevent potential patients turning away from what might be an effective treatment, it fails to address the complex role of therapists’ ontological assumptions in shaping their patients’ experiences. The field must grapple with the tension of holding onto the uniqueness of these ineffable experiences including its roots in indigenous practices and underground work; whilst trying to move into the medical and therapeutic mainstream (Williams and Labte, 2019; George et al., 2020; Noorani, 2020; Gerber et al., 2021).

The so-called “Grofian model”, devised by psychiatrist, Stanislav Grof, suggests a stage-wise process via which people first “work through” personal, biographical material prior to moving to the so-called transpersonal realm (Grof, 2008). This model might help explain the stage-wise influence of emotional breakthrough (dose 1) and mystical experiences (dose 2) in the present study’s path models. It is notable that higher baseline depression was associated with less mystical experience but had no impact on emotional breakthrough in the first session. One speculative explanation, for this is that more severely depressed patients often have a more complicated history of adversity that naturally lends itself to a stage-wise process of first working through personal material before progressing to a transpersonal stage. It is worth noting that anecdotal evidence also exists for the idea of an initial mystical experience allowing an opening to more biographical work, and of course psychedelic experiences can contain a multitude of different experiences (Grof, 2008), but these ideas could be tested in the future.

### Specificity

The supplementary path models for the escitalopram arm differed from those of the psilocybin arm in most respects, serving to support the specificity (to psychedelic assisted-therapy) of the main models presented above. A stronger relationship between depression severity at baseline and endpoint was observed for the escitalopram arm, compared to the psilocybin arm. This indicates that treatment responses after psilocybin treatment are less dependent on the severity of baseline symptoms and that a larger extent of variability in final outcomes is explained by treatment-specific variables such as acute subjective effects and the therapeutic relationship compared to treatment with SSRI’s. Baseline depression also accounted for a greater amount of the explained variance in the escitalopram arm. The therapeutic relationship (therapeutic alliance or rapport) did not predict either the acute experience or final outcomes in the escitalopram arm, indicating that the therapeutic relationship was of greater clinical importance in the psilocybin arm. There was non-significant effect from the acute experience (EBI and MEQ) on final outcomes in the escitalopram arm.

### Strengths and Limitations

This is the first study to provide quantitative evidence supporting the importance of the role of the therapeutic relationship in shaping both the nature of the acute psychedelic experience and subsequent treatment outcomes in a clinical trial of psychedelic-assisted therapy. It is also the first study to validate the importance of emotional breakthrough in mediating therapeutic outcomes in a clinical population or trial.

As pointed out previously (Baier et al., 2020; Kangaslampi, 2020), the nascent field of mechanistic studies in psychedelic research suffers from several methodological limitations, including the lack of appropriate testing of putative causal relationships. By implementing sequential mediation analyses on variables that were measured across successive time points, the current study was able to inform on causal relations in the treatment process. Importantly however, the path analyses applied here, usually require large sample sizes to be considered reliable (Kline, 2015); although rules-of-thumb for sample size estimations in structural equation modelling have been found to be insufficient (Wolf et al., 2013). An important limitation of the current study therefore arises from the small sample size available for the present analyses. Even though models consistently converged and showed acceptable fit, which is normally underestimated in small samples (Ullman, 2001), it is possible that the established parameter estimates are biased by participant characteristics specific to the trial. Future studies should therefore focus on replicating the causal pathways found here in larger samples from psychedelic trials.

One should also note that the present sample was a specific population of individuals with current major depressive disorder. Therefore, we caution against extrapolating to other populations. All participants in this trial were under primary care (i.e. their GP) and were able to demonstrate an ability to build rapport, deemed necessary because of the constraints of the trial design. It is important to consider that many patients with chronic depression have significant comorbidities and complexity (Westen et al., 2004). The demographic in our trial was one typical of clinical research - predominantly white, male and university educated - which does not represent the diversity of patients suffering from depression in the United Kingdom or those most vulnerable to depression (Public Health England, 2019). Lack of diversity and minority exclusion in psychedelic research is a recurrent issue which needs to be addressed (George et al., 2020; Williams and Labate, 2019). Unfortunately, no demographics are available for those who applied to participate (information which should be collected going forward) but the experience of the team was that there was a notable lack of diversity in those applying to participate. Future research should consider diversifying clinical teams, specific culturally-sensitive and targeted recruitment strategies, and appropriate training (Williams et al., 2020). Ongoing and planned studies from the research group have been utilising Patient and Public Involvement (PPI) to address these issues (Close et al., 2021). Furthermore, stigma in relation to sexuality, gender or race has been shown to negatively impact the therapeutic alliance, especially when therapists are not appropriately trained or multiculturally competent (Anderson et al., 2019; Williams et al., 2021), an area future research should explore.

A finding worth reflecting on is the low correlation between therapeutic alliance prior to the first psilocybin session and the acute experience of that session (see Supplementary Table S3). This may be a spurious finding as measures of rapport prior to the first psilocybin session correlated highly with measures of alliance and the acute experience; nevertheless, it is worth considering some possible implications of this. The STAR-P, used here to measure therapeutic alliance, speaks to ideas of the working relationship between patient and therapist (McGuire-Snieckus et al., 2007). Hence it may be that data on this aspect of the therapeutic relationship was less accurate initially, since participants had only three clinical contacts with their therapist at this point, only two of which were in-person. Perhaps the most constructive interpretation here with regards to future research is that pre-session rapport (measured shortly before dosing) may be a more informative predictor of the nature of session one (and thus, longer-term outcomes) than an earlier measure of therapeutic alliance. The downside of this, however, is that pre-emptive action is arguably easier to take at an earlier stage in a process than in the minutes prior to dosing itself. Beyond psychedelic therapies, measures of mean therapeutic alliance across psychotherapeutic sessions has been shown to be a stronger predictor of outcome than early alliance alone (Krupnick et al., 2006).

The primary endpoint of this trial was at 6 weeks, so the present results are only relevant to this relatively brief window of time. It is possible that findings may change, if we were to include longer-term follow-up assessment results in our models or more regular sampling of therapeutic alliance and rapport. Indeed, critics of the proposed 2017 (now delayed to 2022) National Institute of Health and Care Excellence (NICE, 2009) guidelines for depression pointed to the inadequacy of short-term outcome data for informing on long term and persistent conditions such as depression (McPherson et al., 2018).

Another important limitation of the present work was that we did not assess countertransference or clinician rated therapeutic alliance, i.e., the feelings of the guides towards their participants. This might not be considered a major limitation as most evidence suggests that the patient’s perception of the alliance is as good as, if not a more accurate predictor of outcomes (Baier et al., 2020). However, future research might consider including relevant assessments in order to compile a more complete, bidirectional picture of the therapeutic relationship. Assessments of individuals therapists’ ontological assumptions might also yield interesting findings, such as how they may unconsciously influence outcomes. Furthermore, the therapy manual has not been peer reviewed.

### Clinical Implications and Future Directions

These findings highlight the importance of the therapeutic relationship in the psychedelic-assisted therapy treatment process. Development of the required skills for psychedelic-assisted therapy requires appropriate training and experience. Aspects of the therapeutic relationship are likely to be intensified in this work; meaning that in our opinion understanding of ideas such as transference and countertransference dynamics, enactments, boundaries and working with endings is vital; especially when working with clinical populations. Most psychotherapy training programmes require that training psychotherapists undergo their own personal work, often receiving psychotherapy themselves as part of the training process. Future research might consider assessing the impact of the guide’s professional and personal experiences of both standard therapy and of non-ordinary states on clinical outcomes. Our results indicate that factors such as the expertise of the clinical team, and the flexibility and design of the clinical protocol should be carefully considered when deciding to work with participants who may find it more challenging to build a strong therapeutic alliance and rapport. The guides involved in the present trial all received supervision from an experienced psychotherapist, who had extensive experience with therapy in the context of non-ordinary states of consciousness. It is our opinion, that as in traditional psychotherapy, supervision with an experienced clinician is invaluable in order to support high quality therapeutic practice. These and related principles are broadly reflected in published training programmes for psychedelic-assisted therapy (Mithoefer et al., 2017; Phelps, 2017; Tai et al., 2021).

In the United Kingdom traditional psychotherapies are delivered both privately and in the NHS, and so psychedelic-assisted therapy could function as an occasional catalyst to such practices, e.g., to resolve therapeutic impasses, and empirical evidence - such as our results here - may help clinicians and services decide on patient selection and when in a treatment process they might consider a psychedelic experience. Our results could suggest that, within the context of this particular trial design and therapeutic framework, one might consider delaying a dosing session until a sufficiently strong therapeutic alliance is in place. This may apply either before the first or any subsequent dosing sessions. The present findings also suggest that if someone has not responded to the first session (here indicated by low EBI and MEQ scores and high QIDS) that a second session may not confer any additional benefit. One could also infer from the minimal variance explained by the second dosing session, and the direct effects of therapeutic alliance on final outcome, that longer time frames with more therapy between dosing sessions could be considered. Questions of how much preparation, how many dosing sessions, how far apart, and with how much integration, are important—and have significant economic implications for developing plans for psychedelic medicine (Carhart-Harris et al., 2021b). Further research comparing different designs is needed to explore these matters.

It has been suggested that the field of psychotherapy in general should move its focus away from outcome to “process-outcome” research, focussing on specific processes and mechanisms of change (Cohen and DeRubeis, 2018; Yakeley, 2014). Currently, much of the focus of psychedelic-therapy research is understandably on evidencing efficacy and safety in well-controlled and standardised trials, but questions of *who* can benefit most, and *how,* are important for the field, especially when working with clinical populations. Qualitative data and case reports may do well to focus on non-responders in order to better understand how to improve treatment for these individuals going forwards. Observer ratings of the therapeutic relationship e.g., on filmed exchanges for training purposes, may be worth exploring, and case studies and qualitative analyses could also be used to good effect. Future research should include consideration of specific patient factors such as attachment style, defensive organisation, and the quality of object relations, as these factors may underlie a person’s ability to form a strong therapeutic alliance; to feel safely contained in order to surrender to a psychedelic experience and the vulnerabilities it may open up; and to safely work through feelings evoked by the ending of the therapeutic relationship post-trial.

Relatedly it is worth considering the limitations of standardised measures and fixed time frames, i.e. a rapid change in QIDS scores, in capturing the complexity of psychological change and the therapeutic process. It is well established that addressing and working through emotionally challenging biographical material may result in a worsening of symptoms in the short-term but may ultimately enable a deeper understanding and connection to oneself and others in the long term (Lemma, 2015; Olson, 2021). As discussed, clinicians need to be mindful of the idealisation of this new treatment and those that offer it, and the impact this might have on the therapeutic process, tempering unrealistic expectations, and working with the disappointment that may follow as the reality of a more long-term process of healing is recognised if difficulties remain or re-emerge. In our trial, we suspected that many of the participants in this trial would have benefitted from further integration or therapeutic work. Indeed, many went on to work with private psychedelic integration therapists or psychedelic integration groups after the core trial period. This would again indicate a need for more flexible treatment protocols with longer-term follow-up and support.

## Conclusion

In this paper, we have found evidence of an effect of therapeutic alliance and rapport on the quality of the psychedelic experience, which in turn was associated with changes in depressive symptom severity 6 weeks later. More specifically, improvements in depressive symptom severity were more strongly affected by emotional breakthrough experiences during the first, and mystical-type experiences during the second session, respectively. The strength of the therapeutic relationship after the first, but before the second psilocybin session, predicted final depression scores directly, with better alliance predicting lower eventual depression scores. Higher levels of emotional breakthrough during the first psychedelic session was a predictor of therapeutic alliance at this critical intermediate stage, implying the existence of early prognostic predictors that could inform treatment adaptation at any early stage of the therapeutic process. Importantly on the converse, a weaker alliance led to less emotional breakthrough or mystical experiences, and lower depression scores. Further qualitative and quantitative research will be needed to inform on an evidence-based personalized and adaptive, psychedelic-therapy.

## Data Availability

The datasets presented in this article are not readily available due to human subjects being involved. Requests to access the datasets should be directed to roberta.ni.mhurchu@gmail.com.
