# Supplementary material for: Therapeutic Alliance and Rapport Modulate Responses to Psilocybin Assisted Therapy for Depression
Source: Front Pharmacol. 2022 Mar 31;12:788155. doi: 10.3389/fphar.2021.788155 (PMC9009076; doi:10.3389/fphar.2021.788155)
Supplement: Supplementary file 1 [file DataSheet1.pdf]

frontiers

# *Supplementary Material*

## 1. Supplementary Figures

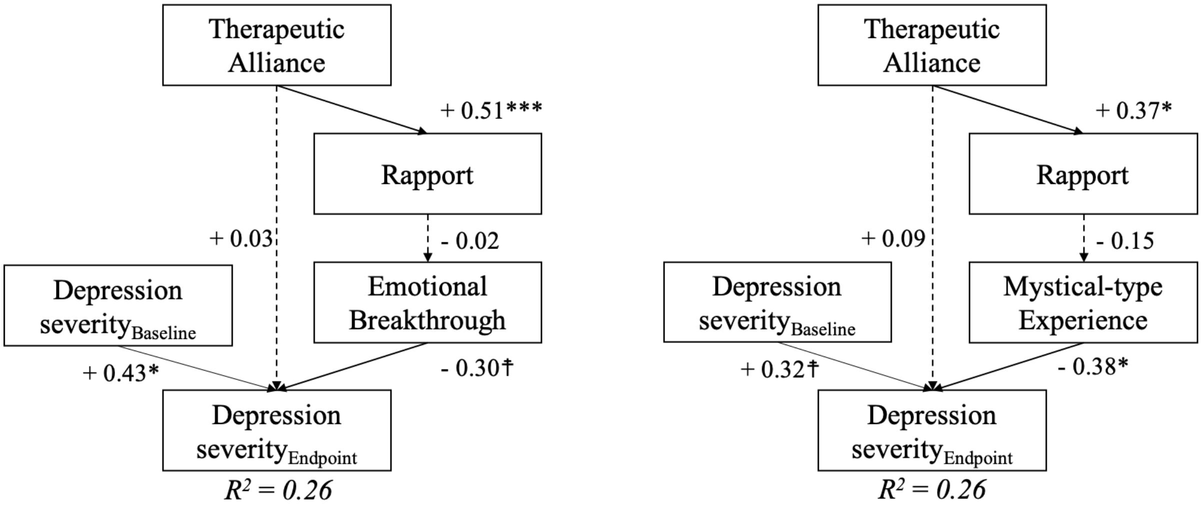


**Supplementary Figure 1**. Path models testing whether therapeutic alliance would lead to better depression scores 6 weeks following the 1mg psilocybin dose administered in the Escitalopram arm of trial. Serial mediation of therapeutic alliance via pre-session rapport and A) Emotional Breakthrough and B) Mystical-type Experiences were not supported by the models, even though acute experience scores still showed trend level significant effects on outcomes. Depression severity at the 6-week Endpoint was adjusted for baseline depression scores - which by itself accounts for $R^2$ = 0.17, i.e., 17% of variance in the final outcome (value not shown on the above models). This means that when this amount of variance was removed, the EBI and MEQ models explained 26% -17% = 9% of variance in final depression scores. Numbers represent standardised regression coefficients for significant (solid) and non-significant (dashed) paths. In comparison, the equivalent path models for 25mg psilocybin the EBI-based model explained 42% - 12% = 30% and the MEQ-based model explained 32% - 12% = 20% of variance in depression severity at the key endpoint

† indicates $p < 0.1$, * $p < .05$, *** $p < .001$

25mg Psilocybin | 1mg Psilocybin + Escitalopram

DD1 EBI

DD2 EBI

DD1 MEQ

DD2 MEQ

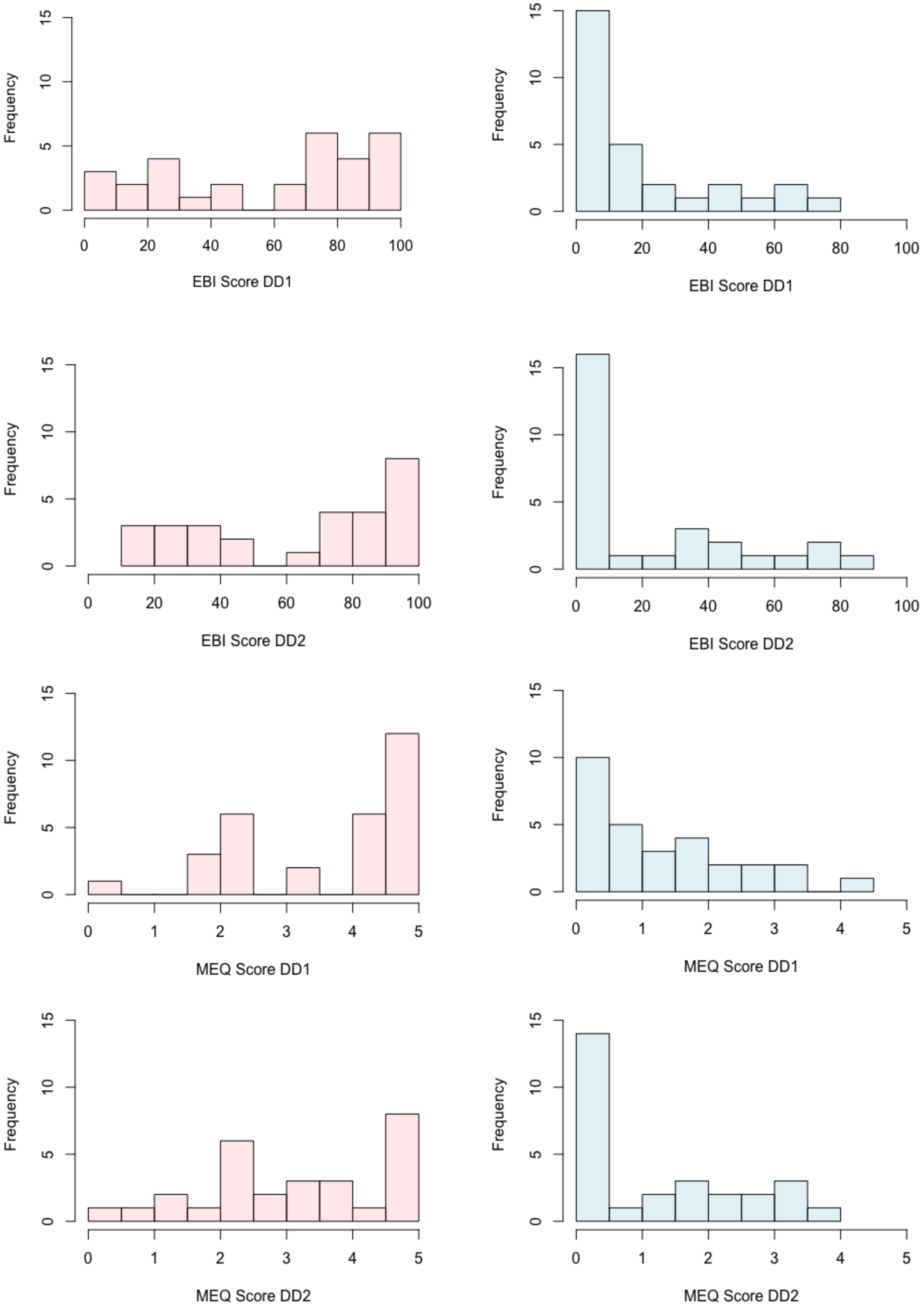

**Supplementary Figure 2**. Distribution of acute subjective experience scores across two dosing sessions with 25mg (left, pink) and 1mg (right, light blue) of psilocybin. MEQ scores displayed as mean scores across all 30 items, where 0 = ‘none, not at all’, and 5 = ‘extreme (more than any other time in my life and stronger than 4)’. MEQ: Mystical Experience Questionnaire; EBI: Emotional Breakthrough Inventory, DD: Dosing day

## 2. Supplementary Tables

**Supplementary Table 1**.

Demographic Information of Participants in the Escitalopram Arm of the Trial

| | |
|---|---|
| **Overall** | |
| Total Patient Number Included in model | 29 |
| Total Patient Number Excluded from model | 0 |
| **Demographics of those included** | |
| Age, years - mean, SD (range) | 39.1, 9.7 (22-60) |
| Females - number (%) | 9 (31) |
| Caucasian Ethnicity - number (%)† | 24 (83) |
| Employment status - number (%) | |
| Employed | 21 (72) |
| Unemployed | 5 (17) |
| Student | 3 (19) |
| University-level education - number (%) | 23 (79) |
| Past psilocybin - number (%) | 8 (28) |
| Weekly alcohol (UK units) - mean, SD (range) | 8.5, 8.3 (0-30) |
| **Clinical** | |
| Illness duration, years - mean, SD (range) | 15.1, 11.0 (2-46) |
| HAMD- 17 scores at pre-treatment baseline - mean, SD (range)¶ | 18.4, 3.4 (11-24) |

| | |
|---|---|
| QIDS-16 scores at pre-treatment baseline - mean, SD (range)§ | 16.4, 4.1 (6-22) |
| No. past meds - mean, SD (range) | 1.8, 1.5 (0-5) |
| Discontinued psychiatric medication for trial - number (%) | 12 (41) |
| Past psychotherapy - number (%) | 26 (90) |

Pre-treatment baseline was 7 to 10 days before dosing-day 1.
† Race was reported by the patients.
§ The scores on the 16-item Quick Inventory of Depressive Symptomatology–Self-Report (QIDS-SR-16) range from 0 to 27,
with higher scores indicating greater depression.
¶ The scores on the 17-item Hamilton Depression Rating Scale (HAM-D-17) range from 0 to 50, with higher scores indicating
greater depression. At screening, which was typically a few weeks before pre-treatment baseline, all the patients
had a score of at least 17 on the HAM-D-17. The depression scores reported in this table are from pre-treatment baseline
and not screening.

**Supplementary Table 2**.

Recommended and actual fit indices for confirmatory path models

| | Psilocybin | | Escitalopram | | | |
|---|---|---|---|---|---|---|
| Fit index | EBI | MEQ | EBI | MEQ | Good fit | Acceptable fit |
| CFI | .979 | .983 | 1.000 | 0.836 | > .95 | > .90 |
| $\chi^2$ /df | 1.20 | 1.17 | 0.50 | 1.60 | < 2.0 | < 3.0 |
| RMSEA | .088 | .078 | 0.000 | 0.152 | < 0.08 | < 0.1 |

*Note*. Models containing the Emotional Breakthrough Inventory (EBI), and Mystical Experience Questionnaire (MEQ). CFI: Comparative Fit Index, χ2: Chi-square test statistic, df: degrees of freedom, RMSEA: Root Mean Square Error of Approximation

## Supplementary Table 3. Means, Standard Deviations, and Correlation Coefficients

| Variable | *M* | *SD* | 1 | 2 | 3 | 4 | 5 | 6 | 7 | 8 | 9 | 10 | 11 | 12 | 13 | 14 | 15 | 16 | 17 |
|---|---|---|---|---|---|---|---|---|---|---|---|---|---|---|---|---|---|---|---|
| 1. $QIDS_{Change}$ | -8.10 | 5.77 | -- | | | | | | | | | | | | | | | | |
| 2. $QIDS_{BL}$ | 14.69 | 3.86 | -.32 | -- | | | | | | | | | | | | | | | |
| 3. $QIDS_{3W}$ | 7.55 | 5.75 | .37* | .57** | -- | | | | | | | | | | | | | | |
| 4. $QIDS_{Final}$ | 6.59 | 5.83 | .78** | .35 | .74** | -- | | | | | | | | | | | | | |
| 5. $EBI_{DD1}$ | 58.80 | 33.38 | -.44* | -.19 | -.40* | -.56** | -- | | | | | | | | | | | | |
| 6. $EBI_{DD2}$ | 65.09 | 30.79 | -.31 | -.26 | -.52** | -.52** | .68** | -- | | | | | | | | | | | |
| 7. $EBI_{Max}$ | 68.93 | 30.07 | -.43* | -.22 | -.44* | -.57** | .90** | .84** | -- | | | | | | | | | | |
| 8. $MEQ_{DD1}$ | 3.59 | 1.38 | -.28 | -.36 | -.47* | -.52** | .63** | .62** | .71** | -- | | | | | | | | | |
| 9. $MEQ_{DD2}$ | 3.22 | 1.42 | -.35 | -.41* | -.49** | -.66** | .52** | .64** | .62** | .75** | -- | | | | | | | | |
| 10. $MEQ_{Max}$ | 3.76 | 1.32 | -.34 | -.35 | -.47* | -.56** | .68** | .67** | .75** | .97** | .83** | -- | | | | | | | |
| 11. $Rapport_{preDD1}$ | 92.07 | 8.30 | -.21 | -.06 | -.28 | -.25 | .56** | .60** | .63** | .56** | .55** | .58** | -- | | | | | | |
| 12. $Rapport_{preDD2}$ | 93.19 | 9.30 | -.25 | -.18 | -.46* | -.36 | .54** | .60** | .60** | .61** | .57** | .64** | .90** | -- | | | | | |
| 13. $STAR_{DD1}$ | 46.08 | 2.02 | .00 | .19 | .08 | .14 | .13 | .15 | .16 | .11 | .19 | .07 | .50** | .41* | -- | | | | |
| 14. $STAR_{DD2}$ | 43.34 | 5.58 | -.41* | -.22 | -.49** | -.56** | .65** | .40* | .50** | .43* | .42* | .45* | .51** | .57** | .34 | -- | | | |

*Note. M* and *SD* represent mean and standard deviation, respectively. Pearson correlation coefficients are shown, * indicates $p < .05$. ** indicates $p < .01$. Quick Inventory of depressive symptomatology (QIDS) at baseline $_{[BL]}$, midpoint $_{[3\ weeks]}$ and endpoint $_{[Final]}$. The Emotional Breakthrough Inventory (EBI), Mystical Experience Questionnaire (MEQ) and therapeutic alliance (STAR) at dosing day 1 [DD1] and dosing day 2 [DD2]. Pre-session rapport [Rapport] was measured one day before each dosing day.
